# Supplementary material for: Light-induced unfolding and refolding of supramolecular polymer nanofibres
Source: Nat Commun. 2017 May 10;8:15254. doi: 10.1038/ncomms15254 (PMC5436226; doi:10.1038/ncomms15254)
Supplement: Supplementary Information — Supplementary Figures, Supplementary Discussion, Supplementary Methods and Supplementary References [file ncomms15254-s1.pdf]

Supplementary Information

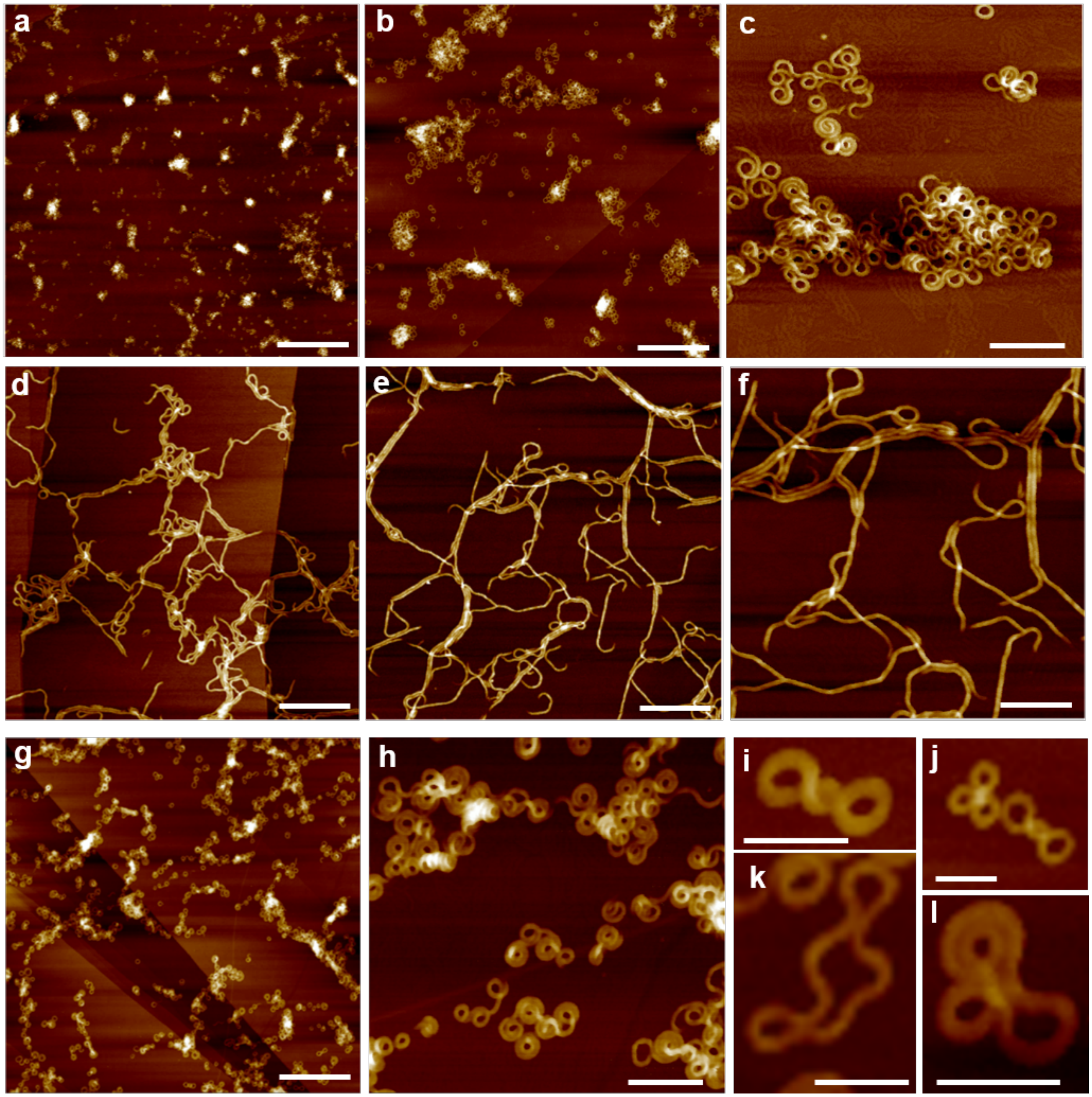

Supplementary Figure 1. Additional AFM images of $\mathbf{SP_{random}}$ (**a**–**c**), $\mathbf{SP_{linear}}$ (**d**–**f**) and $\mathbf{SP_{spiral}}$ (**g**–**l**) with gradual increasing magnification from left to right in each horizontal panel. Scale bars, 800 nm for **a**, 400 nm for **b**, 100 nm for **c**; 400 nm for **d**, 200 nm for **e**, 100 nm for **f**; 400 nm for **g**, and 100 nm for **h**. **i**–**l**, AFM images of various unique macrocyclic supramolecular polymers with spontaneous curvature, which are also observed as a minor amount in the $\mathbf{SP_{spiral}}$ sample. **i**, A twisted macrocyclic supramolecular polymer with two loops that apparently looks like "figure eight". **j**, A twisted macrocyclic supramolecular polymer with three loops (left) and two loops (right). To make these distinctive shapes, the supramolecular polymers changes direction (clockwise/counter clockwise) several times, however, supramolecular polymers maintain their spontaneous curvature throughout the continuous chain. This indicates the merit of spontaneous

curvature to construct supramolecular polymer of unusual shapes with good shape persistency. **k**, A large macrocyclized supramolecular polymer without significant twisting; **l**, An exceptionally macrocyclized supramolecular polymer with an unidirectional continuous up-down trajectory that indicates their formation in solution not in the surface during solvent drying. Scale bars, 50 nm for **i–l**.

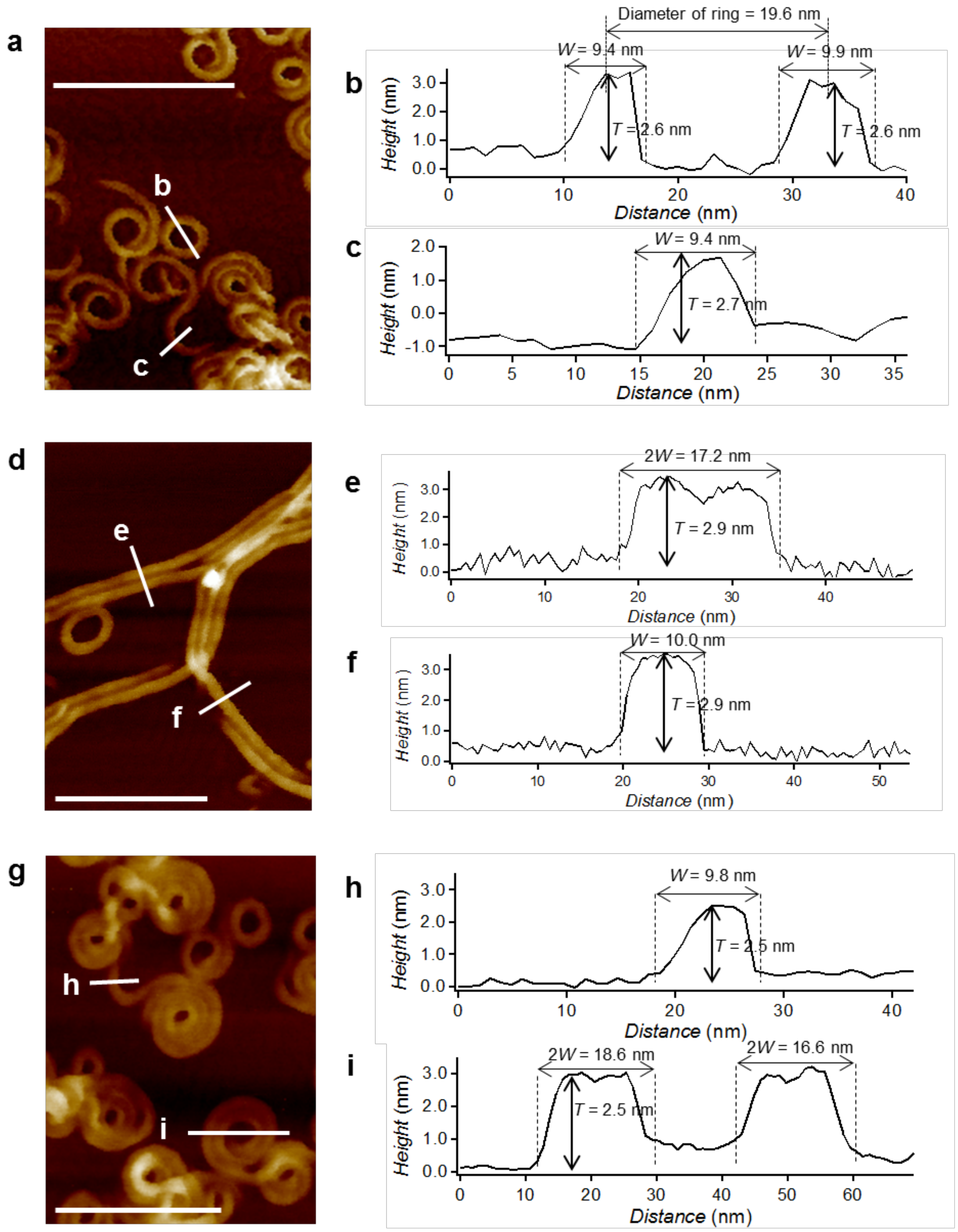

Supplementary Figure 2. AFM cross-sectional analysis of three supramolecular polymers of *trans*-**2**. **a–c**, $\mathbf{SP_{random}}$; **d–f**, $\mathbf{SP_{linear}}$; **g–i**, $\mathbf{SP_{spiral}}$. Scale bars, 100 nm for all. These analyses show that the three supramolecular polymers have similar cross-sectional width (*W*) of ca. 9.7 ± 0.3 nm and thickness (*T*) of ca. 2.7 ± 0.2 nm, suggesting that they are formed based on the same self-assembly process, *i.e.*, tilted stacking of hexamers.

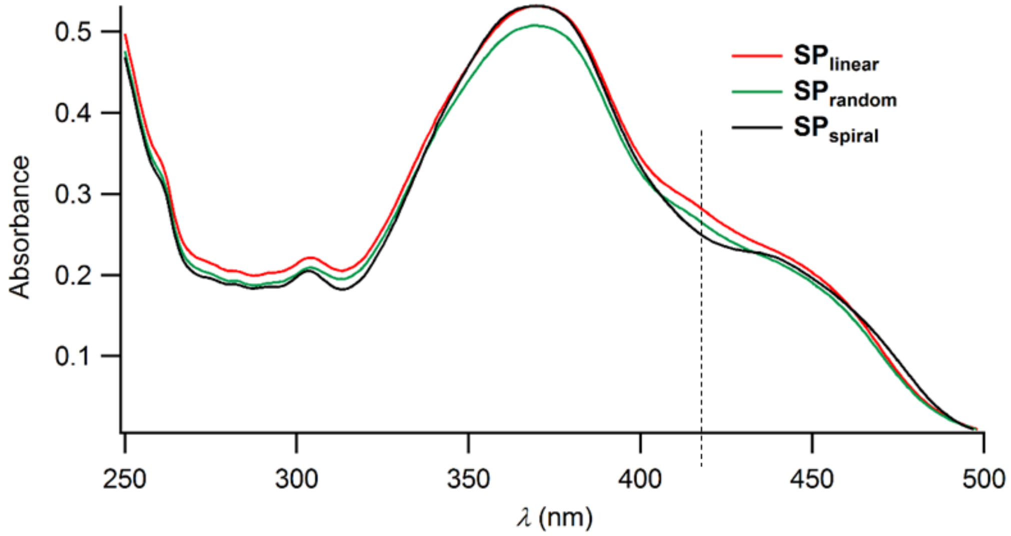

Supplementary Figure 3. UV-Vis absorption spectra of three supramolecular polymers of *trans*-**2** where absorbance at λ = 410–430 nm likely measures level of internal order. The increasing absorbance at λ = 410–430 nm is responsible for increasing disorder. So, degree of internal order $\mathbf{SP_{linear}} < \mathbf{SP_{random}} < \mathbf{SP_{spiral}}$.

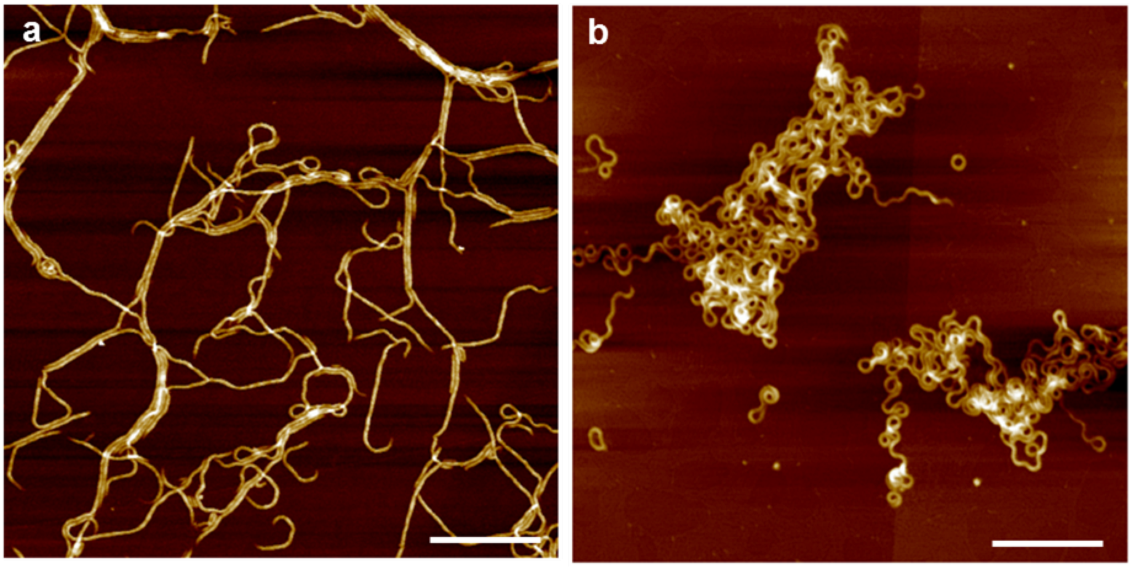

Supplementary Figure 4. AFM images showing spontaneous transformation of $\mathbf{SP_{linear}}$ into $\mathbf{SP_{random}}$ over time. **a**, $\mathbf{SP_{linear}}$ just after quenching; **b**, $\mathbf{SP_{random}}$ obtained from $\mathbf{SP_{linear}}$ upon standing over 20 h.

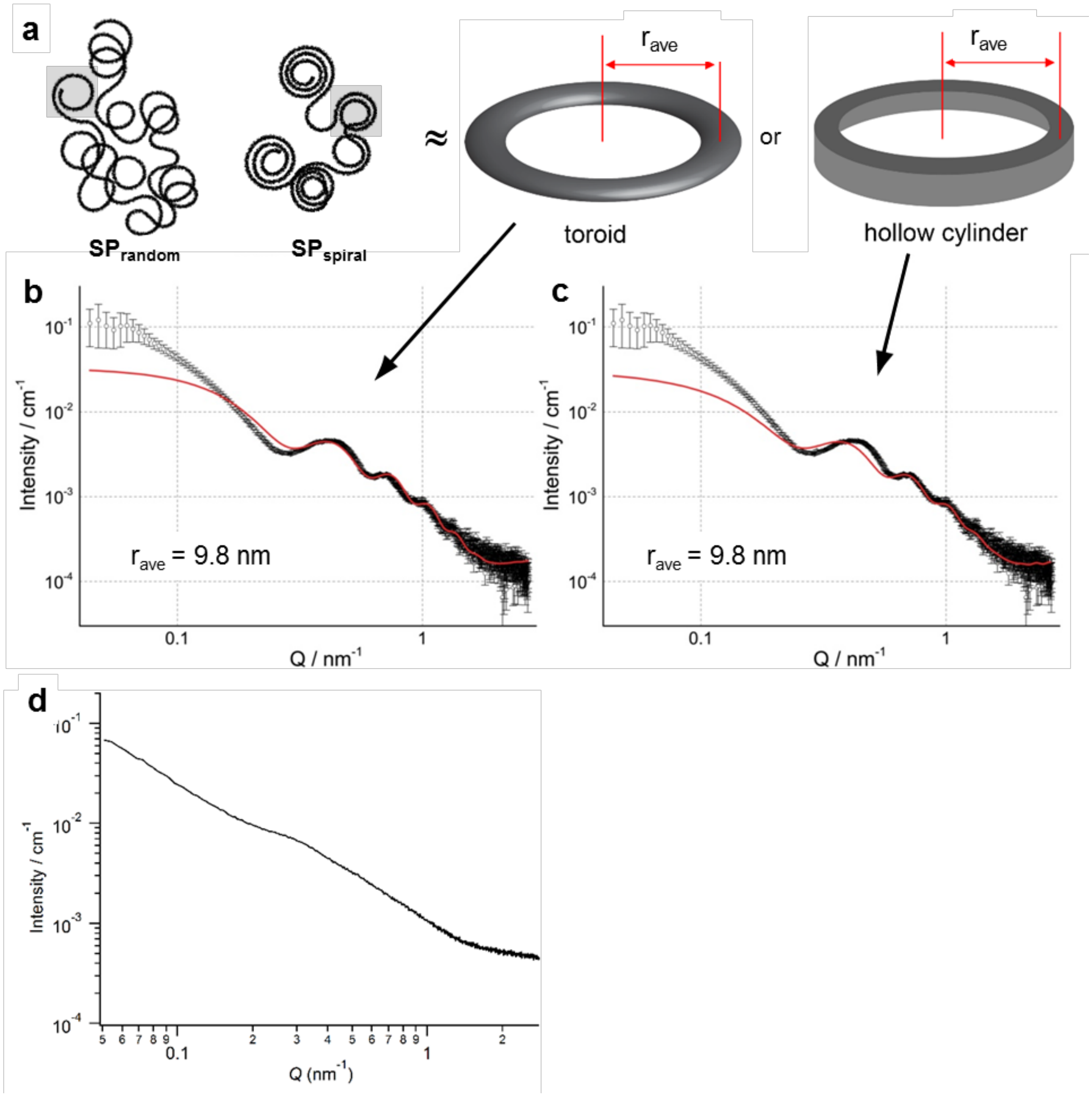

Supplementary Figure 5. a, Shapes used to approximate the looped structures observed in $\mathbf{SP_{random}}$ and $\mathbf{SP_{spiral}}$. **b**, SAXS data for $\mathbf{SP_{random}}$, with toroid model fit (red line) shown. **c**, SAXS data for $\mathbf{SP_{random}}$, with hollow cylinder model fit (red line) shown. Regardless of the model used, the same value of r_{ave} was obtained. **d**, SAXS plots of $\mathbf{SP_{linear}}$ ($c = 1 \times 10^{-4}$ M in MCH) showing absence of maxima/minima.

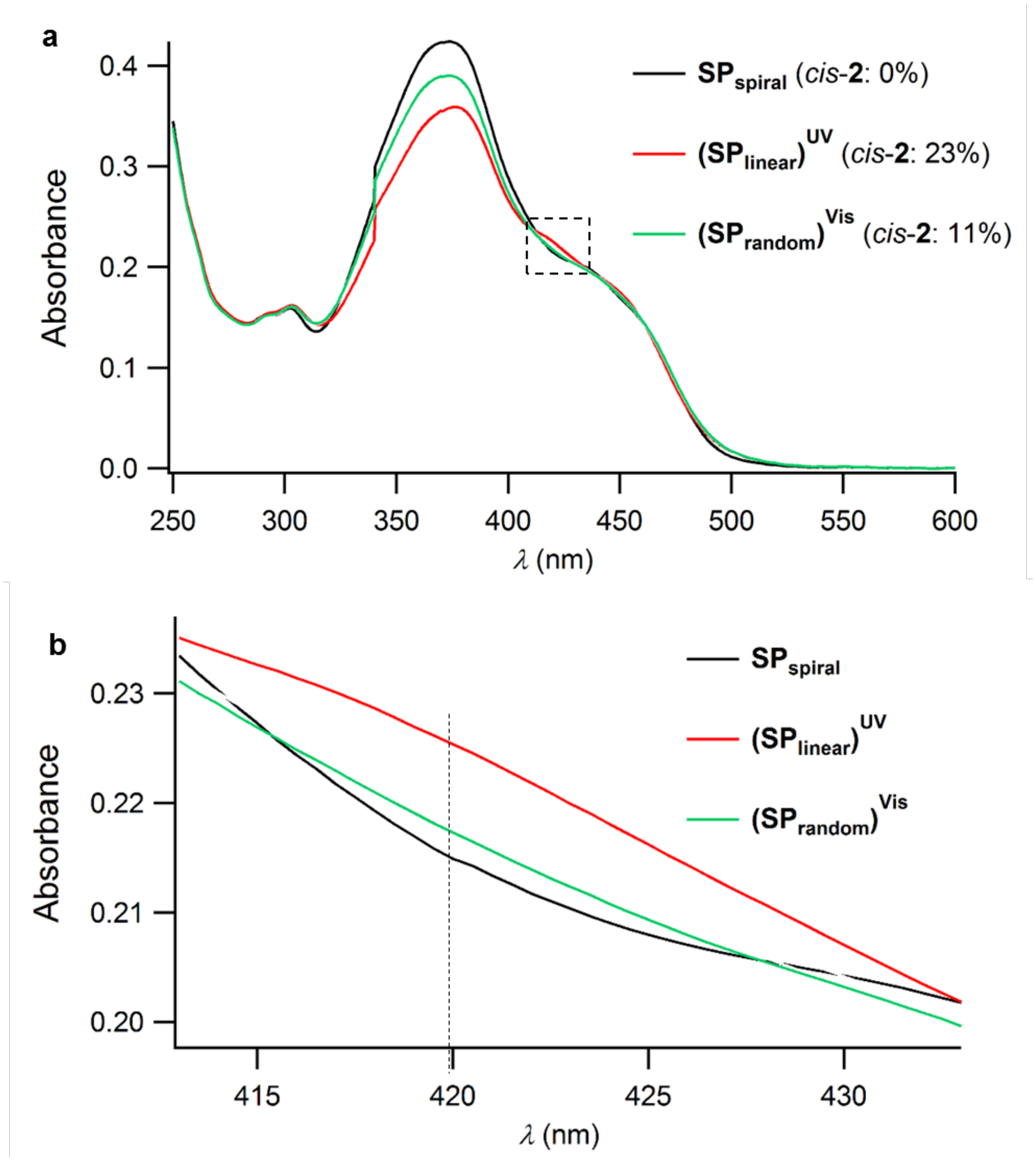

Supplementary Figure 6. a, Full UV-Vis spectra of **2** in MCH ($c = 1.0 \times 10^{-4}$ M, $\mathbf{SP_{spiral}}$) before (black), after irradiation of UV-light (red) and Vis-light (green) subsequently. After UV-and Vis-irradiation, a decrease and subsequent increase in absorbance at λ_{max} were observed, from which the extent of *trans*-to-*cis* and *cis*-to-*trans* photoisomerization of azobenzene moiety could be estimated respectively. **b**, Partial UV-Vis spectra at λ = 413–433 nm which measures degree of internal order. Upon UV-irradiation, an increase in absorbance at 420 nm indicates reduction of internal order which mostly recovers after Vis-irradiation.

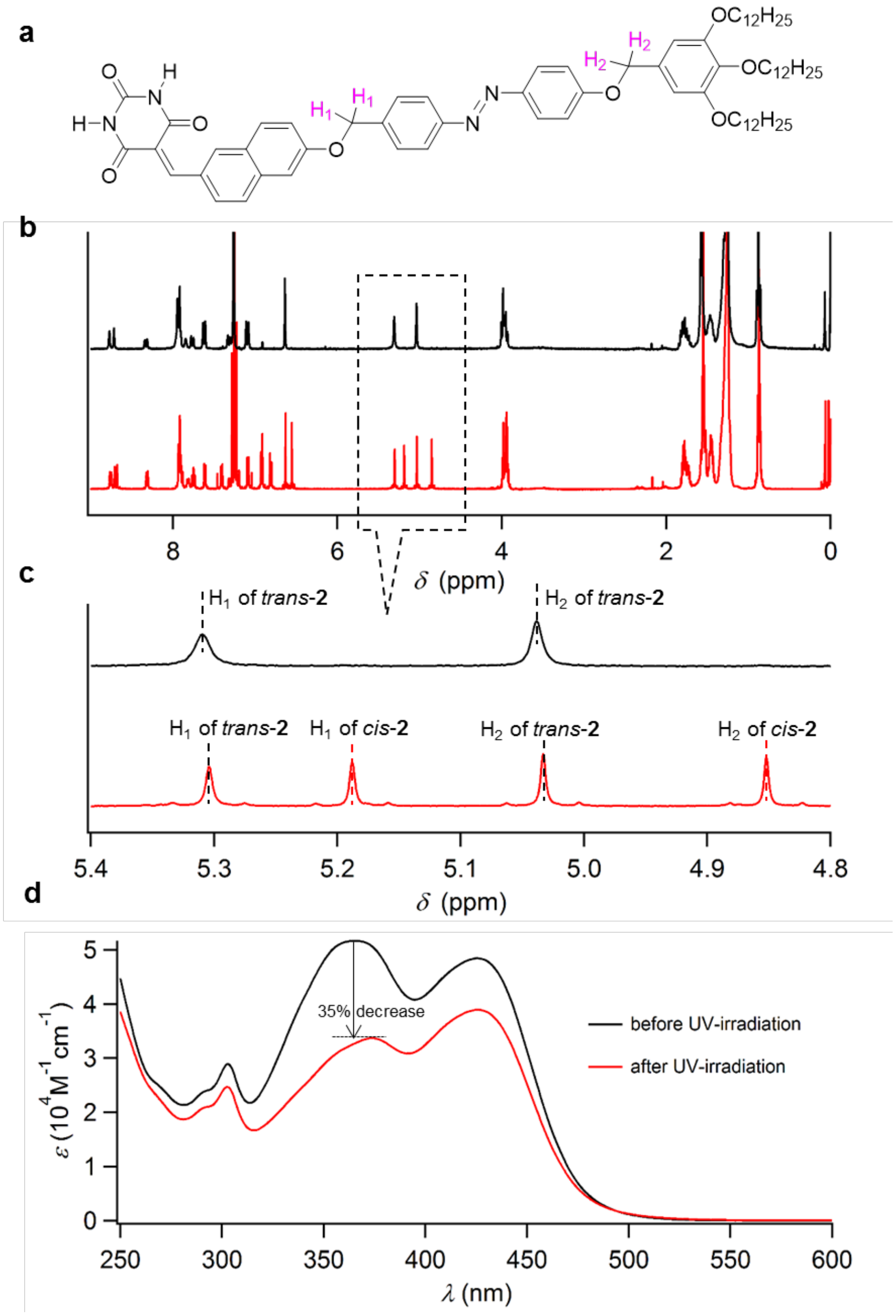

Supplementary Figure 7. **a**, Chemical structure of *trans*-**2** highlighting two sets of benzylic hydrogens (H_1 and H_2) which are susceptible toward photoisomerization. **b**, Full ^{1}H NMR spectra of **2** ($c = 1 \times 10^{-3}$ M) in $CDCl_3$ before (black) and after (red) irradiation of UV-light for 20 min to reach a PSS. **c**, Partial ^{1}H NMR spectra where the integration of the benzylic proton signals of *trans*-**2** and *cis*-**2** suggests that UV irradiation leads to the 50% photoisomerization (*trans*-**2**: *cis*-**2** = 50:50). **d**, UV-Vis spectra of $CDCl_3$ solutions of **2** ($c = 1 \times 10^{-4}$ M) prepared by diluting the solutions used for the above NMR measurements. The maximum absorption intensity of the azobenzene unit (initially at 370 nm) shows 35% decrease upon the UV-irradiation, which corresponds to 50% *trans*-to-*cis* isomerization according to the NMR analysis. This relationship

has been used as a reference to calculate photoisomerization yield of azobenzene unit in supramolecular polymers in MCH.

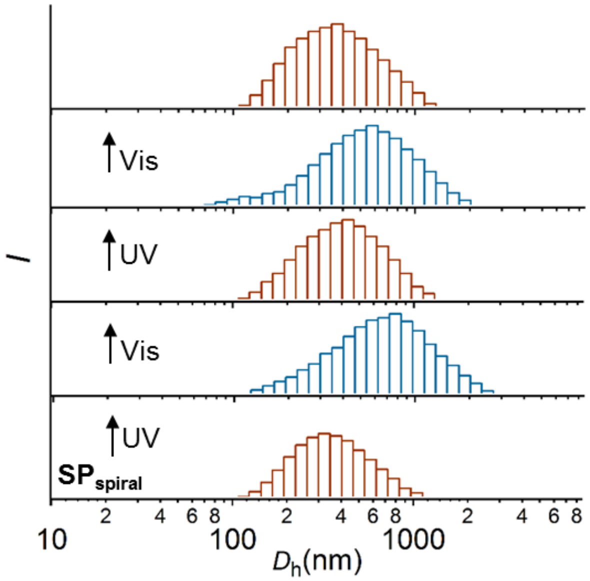

Supplementary Figure 8. DLS analysis of the dilute solution (c = 2.5 × 10^{-5}) of $\mathbf{SP_{spiral}}$ upon successive exposed to UV and Vis light, showing photoinduced changes in the distribution of the hydrodynamic diameters (D_h).

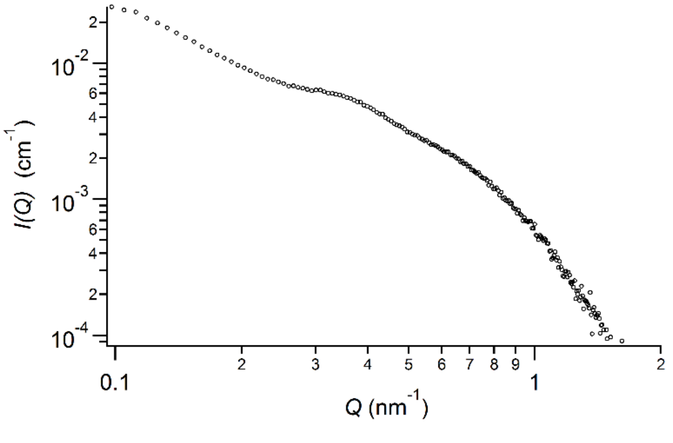

Supplementary Figure 9. SAXS data for $(\mathbf{SP_{random}})^{\mathbf{Vis}}$ with 5% *cis*-**2** content, which was achieved by thermal back-isomerization of the Vis-light irradiated supramolecular polymer solution at dark over 60 h. The plot showed weak contributions from the specific scattering derived from the spontaneous curvature, thus supporting an improved refoldability.

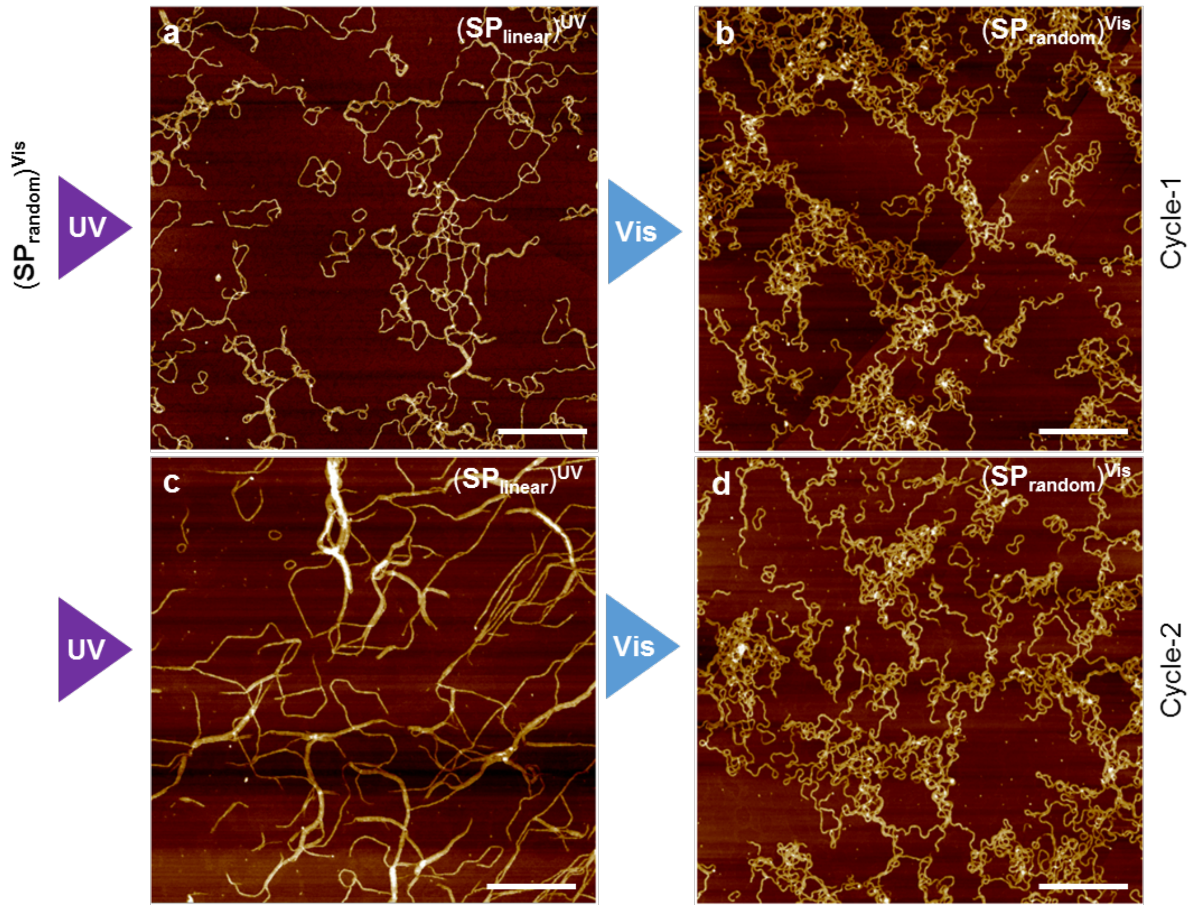

Supplementary Figure 10. AFM images showing a reversibility in the photo-interconversion between (**SP**$_{\mathbf{linear}}$)^{UV} and (**SP**$_{\mathbf{random}}$)$^{\mathbf{Vis}}$. **a**, (**SP**$_{\mathbf{linear}}$)^{UV} obtained by irradiation of (**SP**$_{\mathbf{random}}$)$^{\mathbf{Vis}}$ with UV-light. **b**, (**SP**$_{\mathbf{random}}$)$^{\mathbf{Vis}}$ obtained by subsequent irradiation with Vis-light. **c**, (**SP**$_{\mathbf{linear}}$)^{UV} acquired by succeeding irradiation with UV-light. **d**, (**SP**$_{\mathbf{random}}$)$^{\mathbf{Vis}}$ obtained by subsequent exposure to Vis-light. Scale bars, 400 nm for all images.

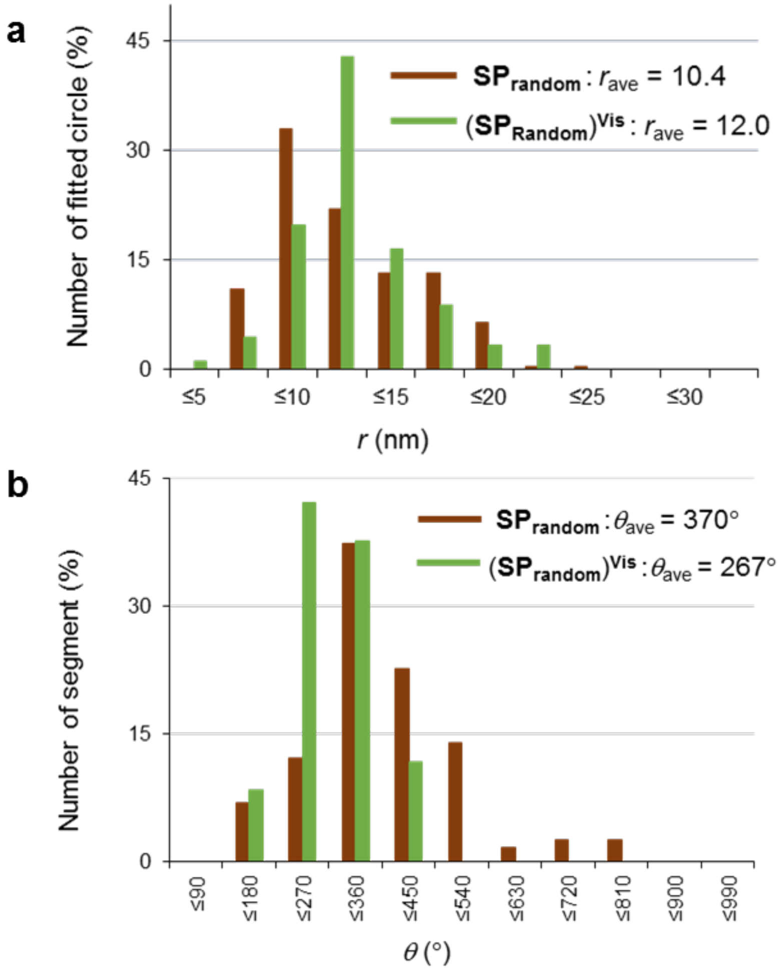

Supplementary Figure 11. a, Comparison of r distribution between $\mathbf{SP_{random}}$ and $(\mathbf{SP_{random}})^{\mathbf{Vis}}$ along with their r_{ave} values. **b**, Comparison of θ distribution between $\mathbf{SP_{random}}$ and $(\mathbf{SP_{random}})^{\mathbf{Vis}}$ along with their θ_{ave} values.

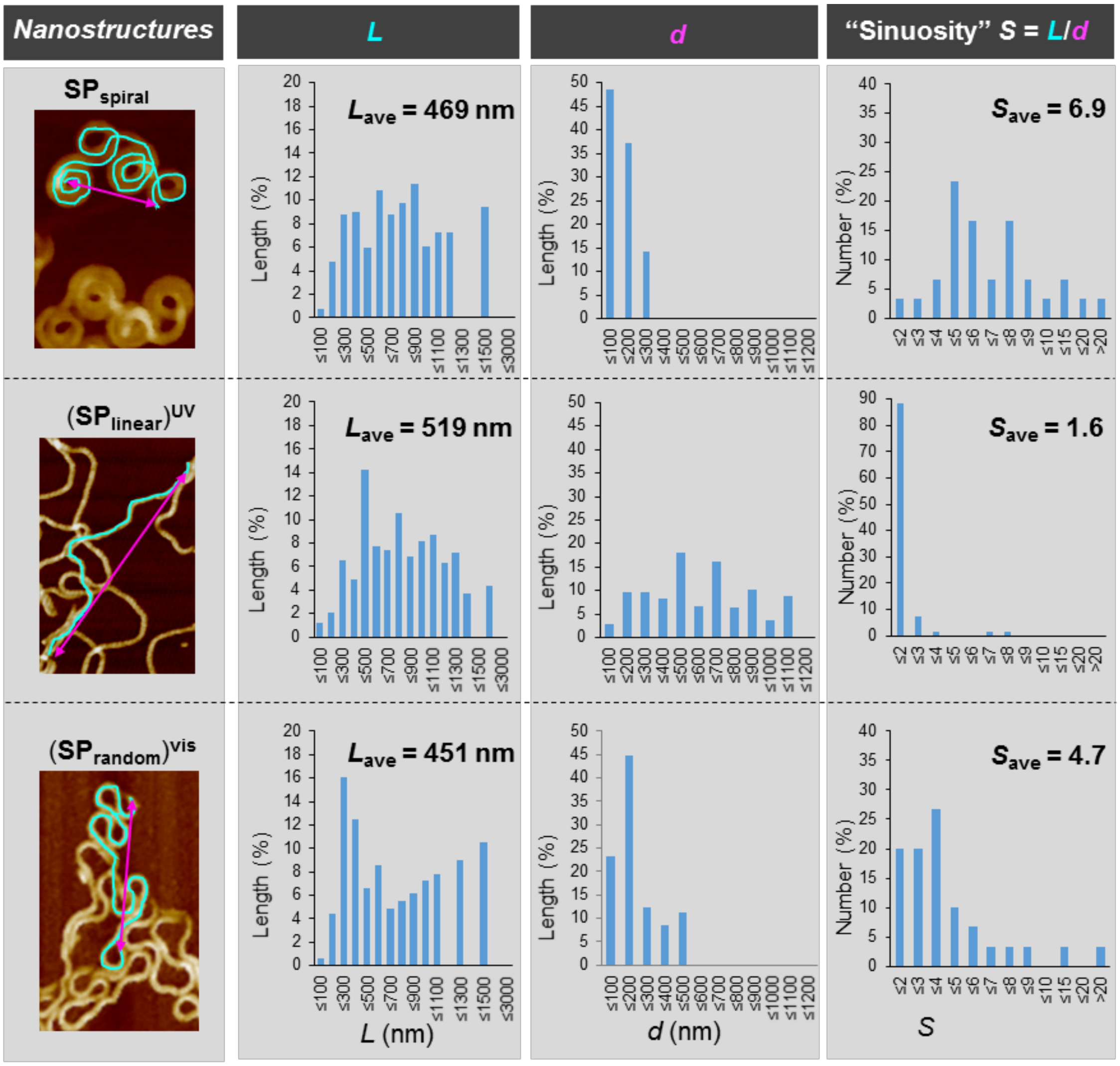

Supplementary Figure 12. Comparison of the entanglement of **SP**$_{\text{spiral}}$, **(SP**$_{\text{linear}}$**)**^{UV}, and **(SP**$_{\text{random}}$**)**^{Vis} according to $S = L/d$ (L = actual path length along the chains; d = the shortest distance; S = sinuosity). In the left panel, AFM images of the three representative SP fibres are traced by one-stroke cyan curves (L) and purple straight lines (d).

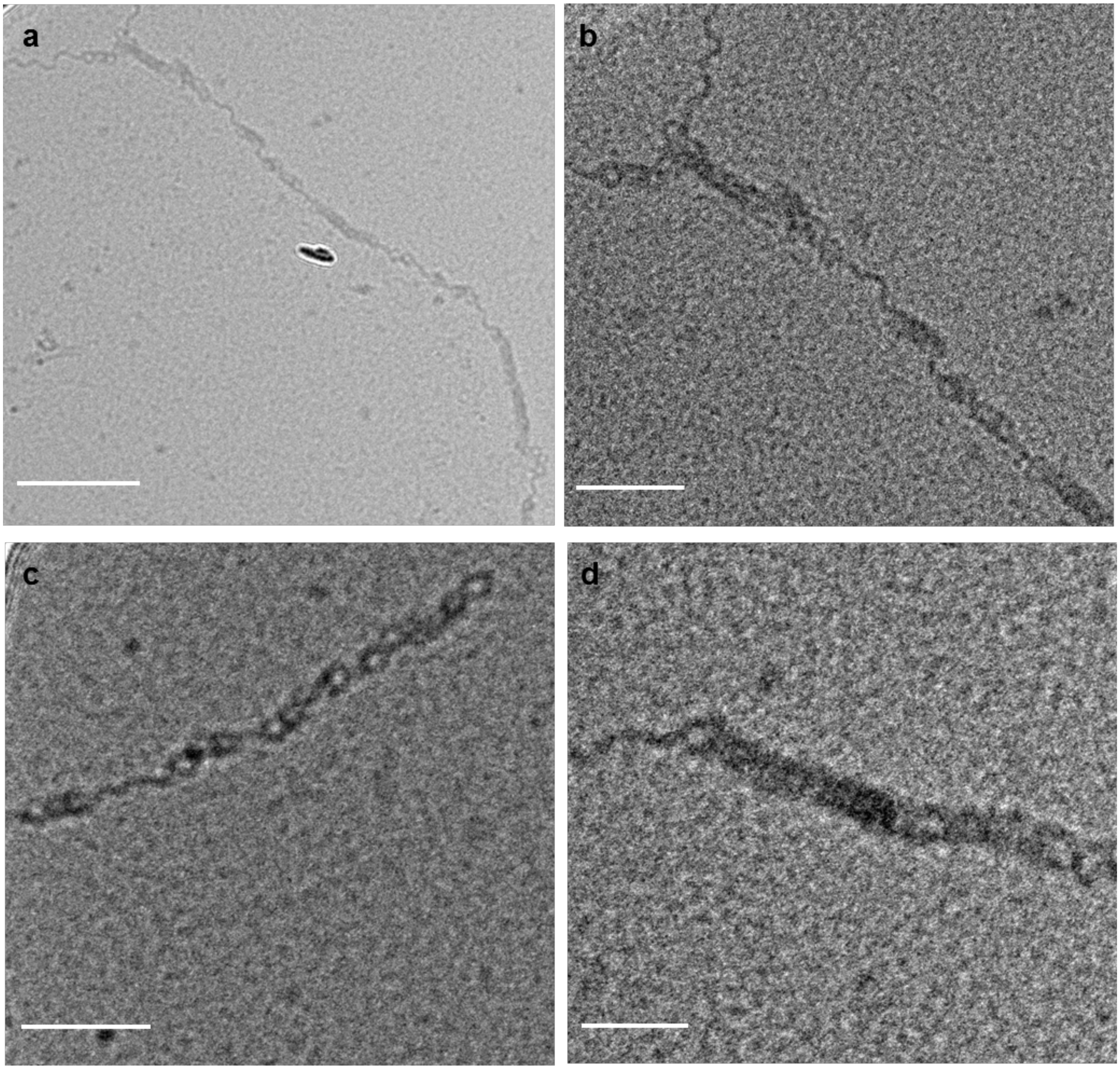

Supplementary Figure 13. TEM images of $\mathbf{SP_{helical}}$. Scale bars, 200 nm for **a**, 100 nm for **b** as well as **c**, and 50 nm for **d**.

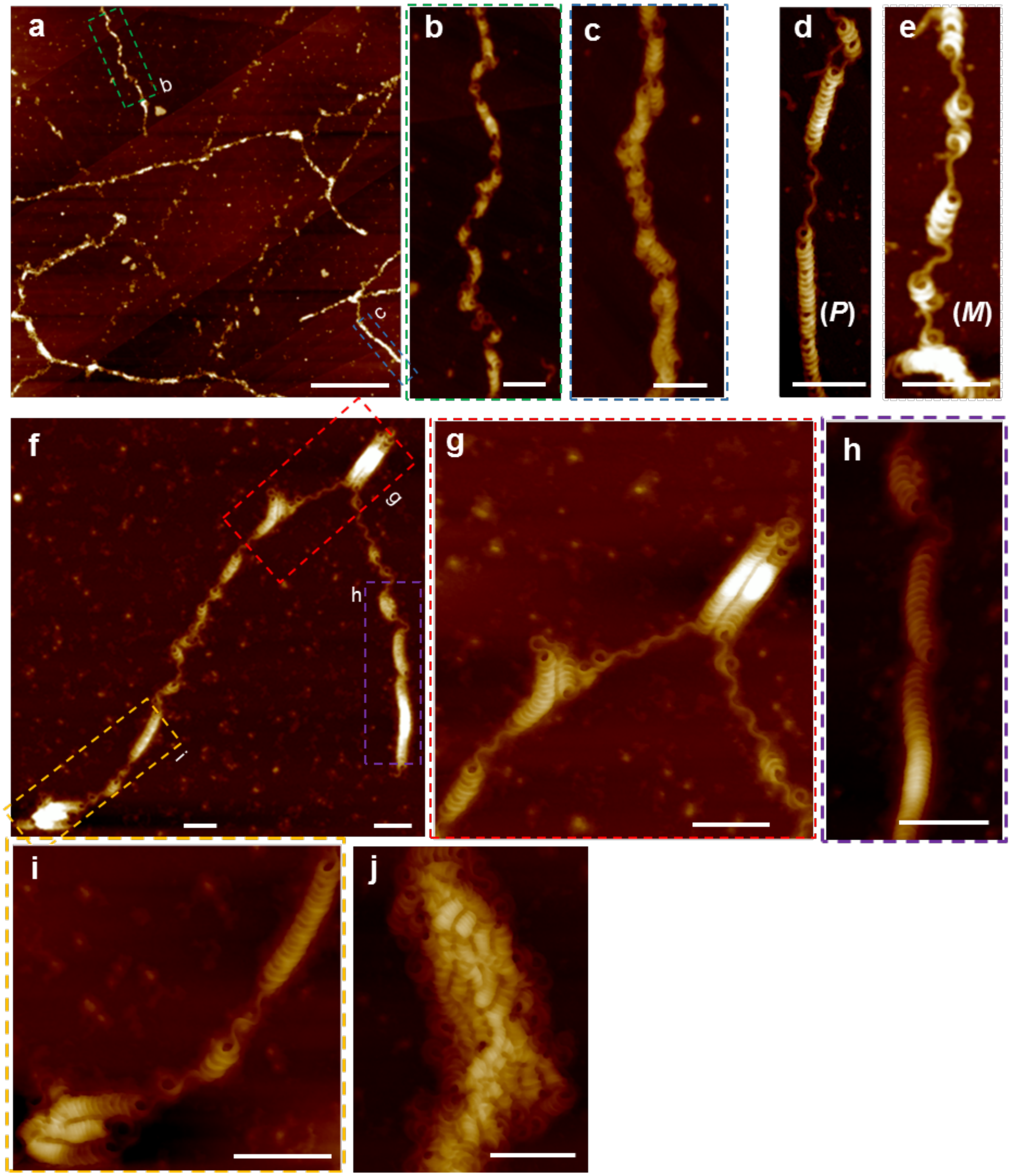

Supplementary Figure 14. Additional AFM images of $\mathbf{SP_{helical}}$. **a**, A large scale image showing a predominant formation of $\mathbf{SP_{helical}}$ at 4 × 4 μm area, where most of the individual $\mathbf{SP_{helical}}$ chains are linearly elongated and bundled up together in a network. **b–c**, Magnified images for two different areas of image **a** as indicated by green and blue rectangles. **b**, A long $\mathbf{SP_{helical}}$ with partially unfolded domains. **c**, A coiled-coil $\mathbf{SP_{helical}}$ where two $\mathbf{SP_{helical}}$ fibres are entangled helically to form a biologically occurring sophisticated secondary structure. **d**, A right-handed (*P*-type) $\mathbf{SP_{helical}}$. **e**, A left-handed (*M*-type) $\mathbf{SP_{helical}}$. **f**, AFM image of $\mathbf{SP_{helical}}$ at 1.5 × 1.5 μm area, where helical handedness is clearly retained throughout the entire fibre despite of the presence of some partially unfolded domain. **g–i**, Magnified images for selected areas in image **f** as indicated

by red, violet and yellowish dotted rectangles. **j**, AFM image showing three-dimensionally highly bundled **SP$_{helical}$**. Scale bar, 800 nm for **a** and 100 nm for all others (**b–j**).

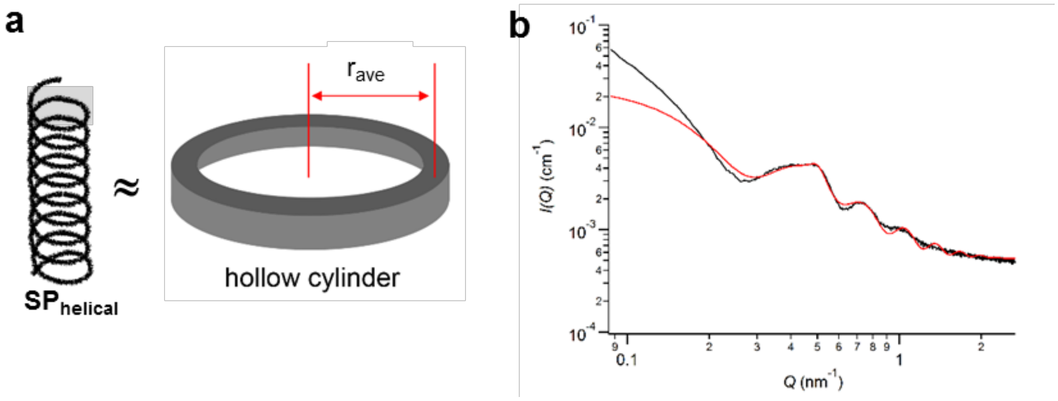

Supplementary Figure 15. a, Shapes used to approximate the looped structures observed in **SP$_{helical}$**. **b**, SAXS data for **SP$_{helical}$** solution ($c = 1.0 \times 10^{-4}$ M in MCH, after evaporation of $CHCl_3$), with hollow cylinder model fit (red line) shown. The r_{ave} was determined to be 10.0 ± 0.2 nm after fitting.

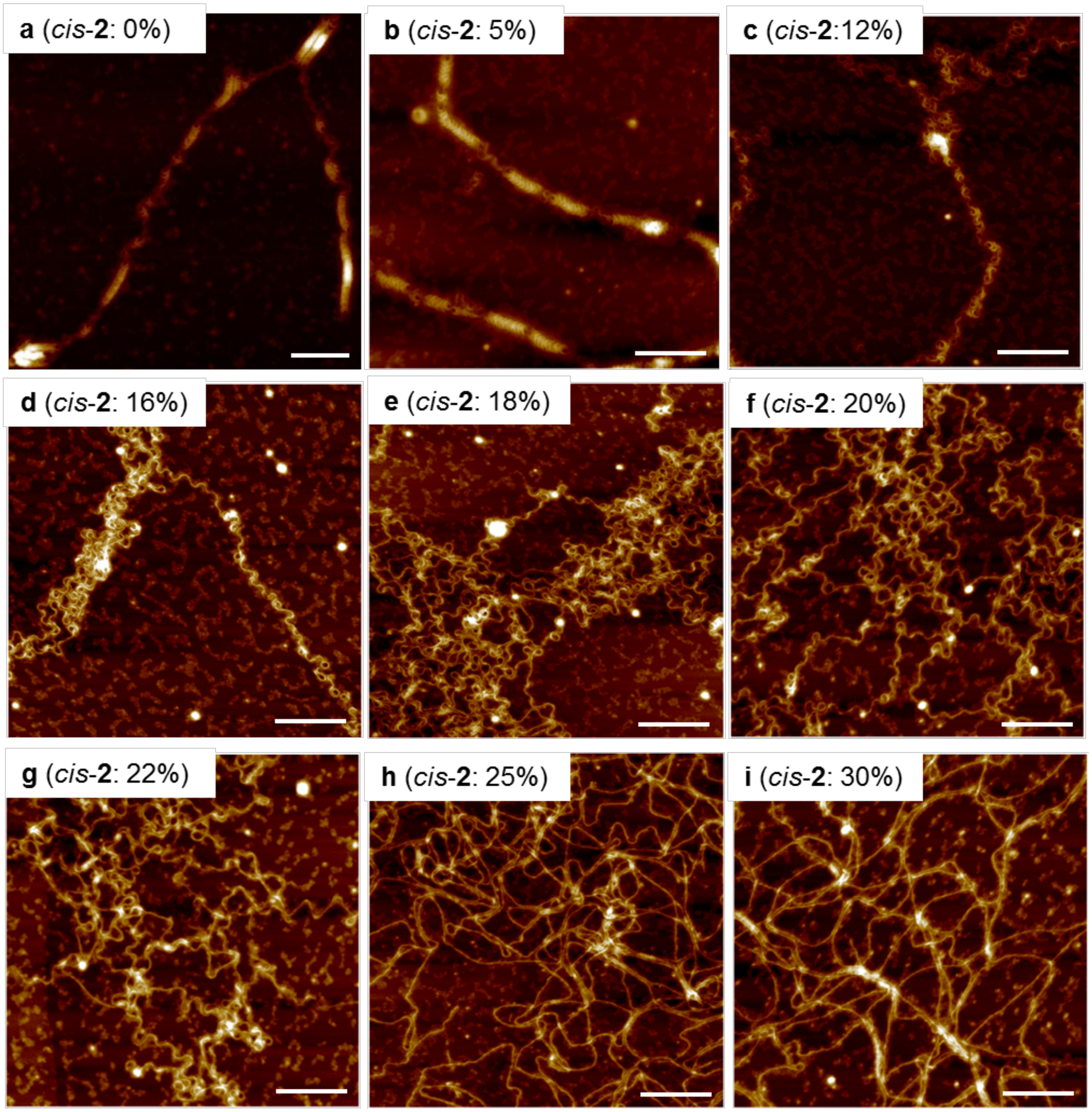

Supplementary Figure 16. a–i, AFM images showing the unfolding process of $\mathbf{SP_{helical}}$ as a function of increasing *cis*-**2** content, which is finely controlled by irradiation with UV light. Scale bars, 200 nm for all.

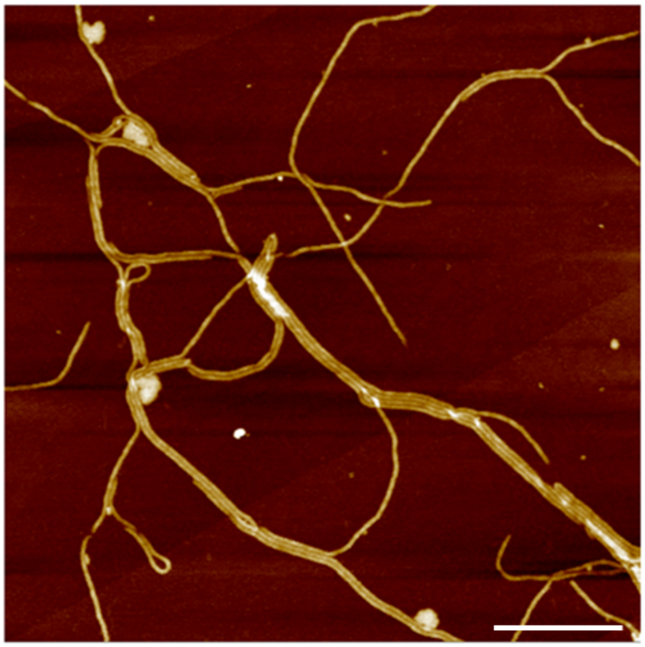

Supplementary Figure 17. AFM image of very straight supramolecular polymer fibres prepared by thermal supramolecular polymerization of *cis*-**2** monomer (in fact a mixture of *trans*-**2** and *cis*-**2**). A hot MCH solution of **2** ($c = 1.0 \times 10^{-4}$ M) at 105 °C ($a_{agg} = 0$) was irradiated with UV-light for five minutes to afford ~40% of *cis*-**2** and then cooled to 20 °C naturally. Scale bar, 200 nm.

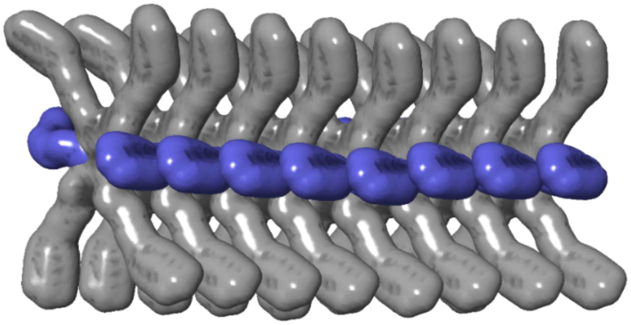

Supplementary Figure 18. A schematic representation for stacking of heteromeric rosettes (involving two *cis*-**2** and four *trans*-**2** molecules) into very straight fibres with long-range domains (~ 500 nm) by aligning all *cis*-arms linearly in space due to their steric demand.

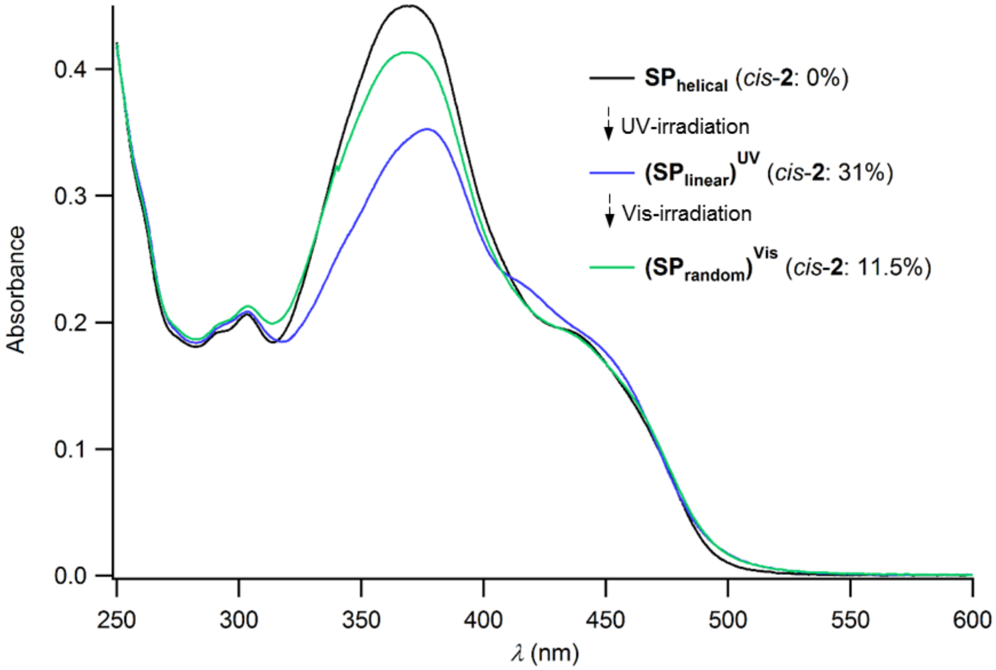

Supplementary Figure 19. UV-Vis spectra of **2** in MCH ($c = 1.0 \times 10^{-4}$ M, $\mathbf{SP_{helical}}$) before (black) UV-irradiation, after (blue) UV-irradiation and subsequent (green) Vis-irradiation. UV-irradiation results in ~31% *cis*-**2**, and subsequent Vis-irradiation (weak Vis-light, 20 cm distance between the light source and sample) takes around 20 minutes to reach PSS. The increase in absorbance at λ_{max} estimates the extent of *cis*-to-*trans* back-isomerization (with 11.5 % *cis*-**2**) of azobenzene moieties in relative to the reference system. It can be noted that at λ = 413–433 nm, an increased absorbance by UV-irradiation, suggest a decrease in internal order of supramolecular polymers, while decreased absorbance by Vis-irradiation indicates recovery of the high degree of internal order of $\mathbf{SP_{helical}}$.

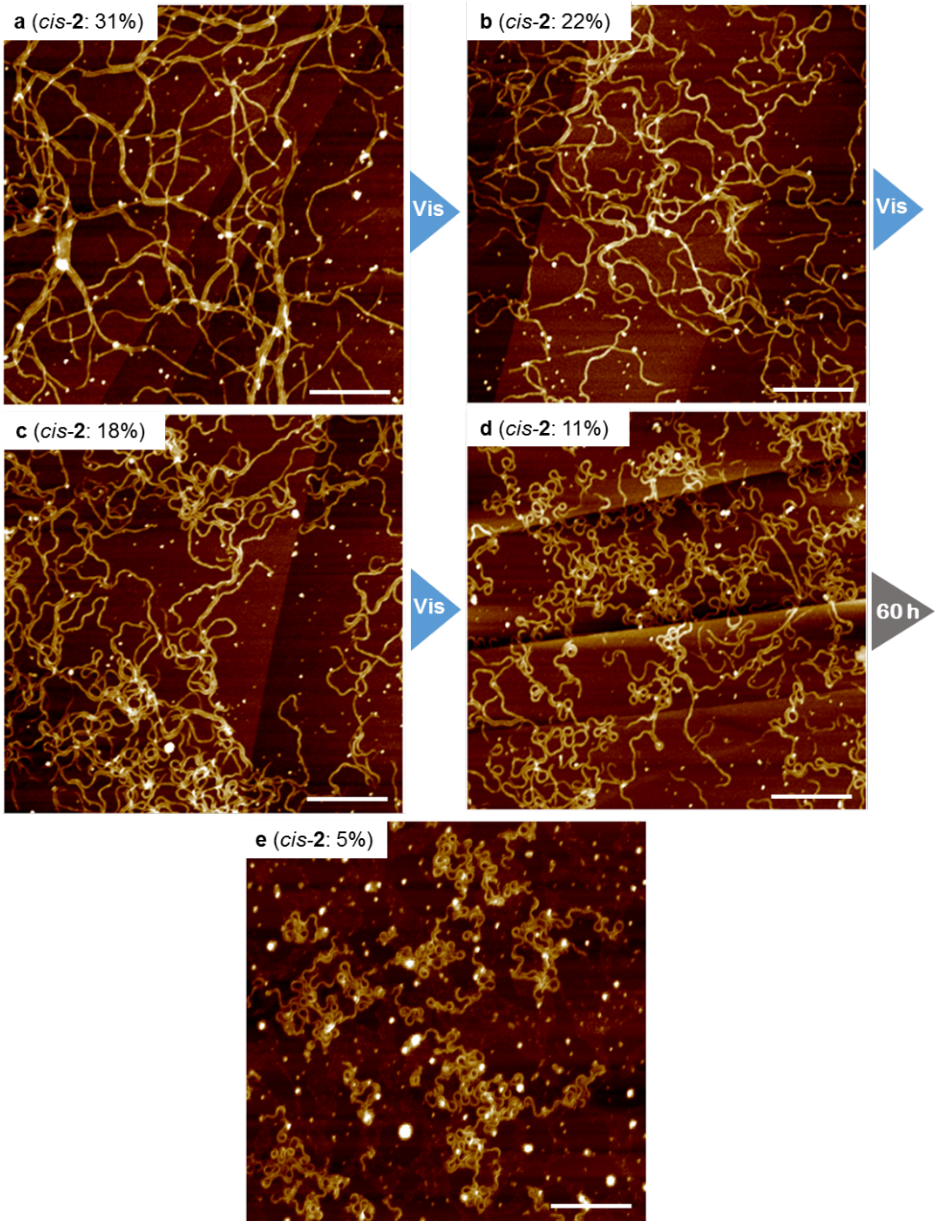

Supplementary Figure 20. a–d, AFM images showing the continuous transformation of $(\mathbf{SP}_{\mathbf{linear}})^{\mathrm{UV}}$ to $(\mathbf{SP}_{\mathbf{random}})^{\mathrm{Vis}}$ upon gradual decreasing *cis*-**2** content controlled by irradiation with weak Vis-light. Exposure to the weak Vis-light is able to linearly recover the spontaneous curvature. **e**, Further decrease in *cis*-**2** content was achieved by standing the supramolecular polymer solution at dark over 60 h. This resulted in further of recovery turning angle (θ), and thus reduction of the dispersity of supramolecular polymers on the surface. Scale bars, 200 nm for all.

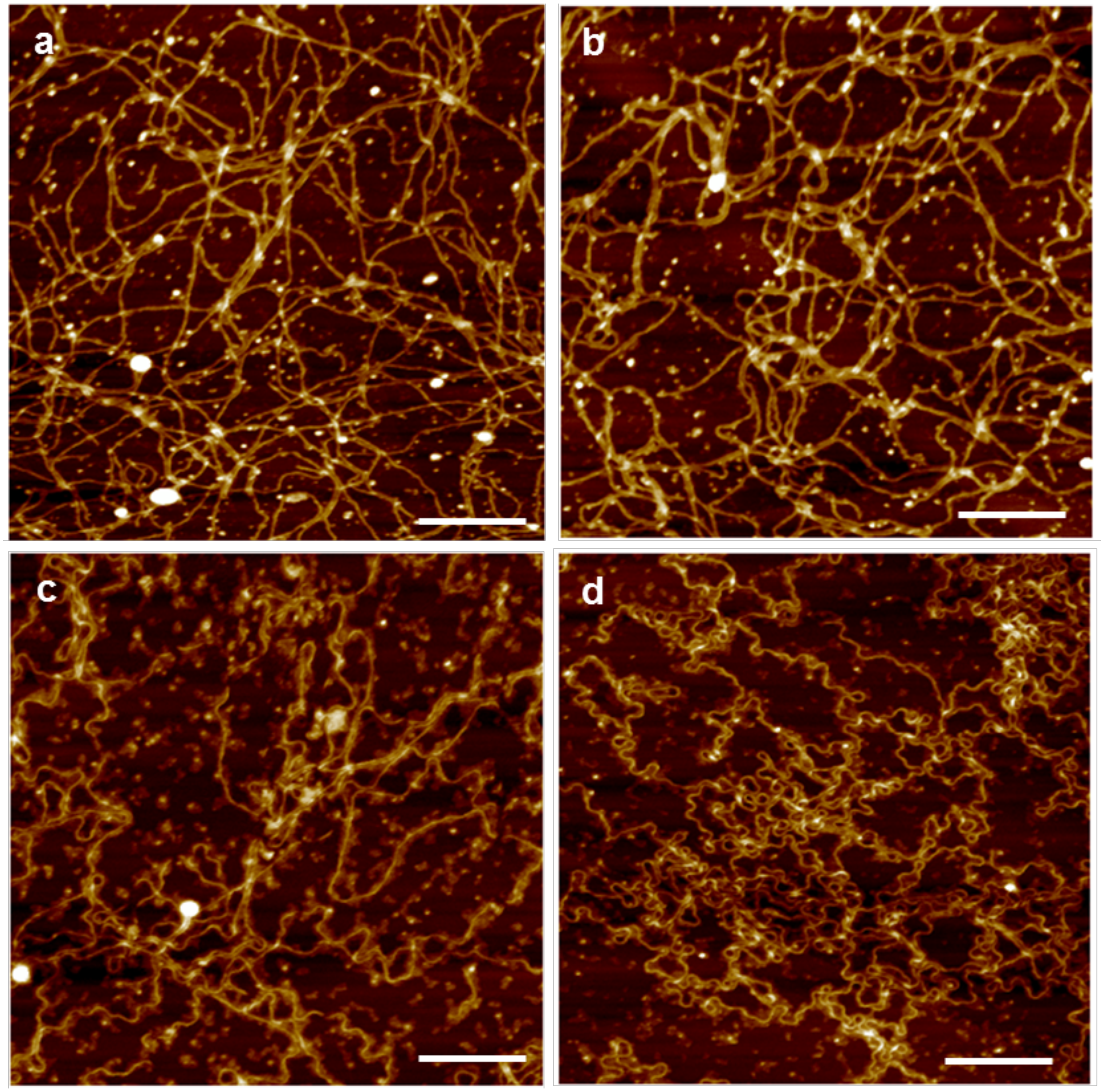

Supplementary Figure 21. AFM images showing a spontaneous conversion of kinetically formed **(SP_{linear})**^{Vis} to (**SP_{random}**)^{Vis} over 20 h. **a**, Just after exposure to strong Vis-light, **b**, After 1 h, **c**, After 5 h, **c**, After 20 h from the irradiation with strong Vis-light for 30 s. This suggests that quick recovery of *trans*-**2** with the strong Vis light is not able to recover the encoded curvature instantly, and it recovers slowly over 20 h.

Supplementary Discussion

Supramolecular polymerization.

Supramolecular polymers of *trans*-**2** were prepared according to the methods shown in Supplementary Figure 22. Supramolecular polymers with variable foldability were prepared by altering cooling rate or adding $CHCl_3$ as co-solvent that can push the supramolecular polymerization process under more thermodynamic conditions. The difference in monomer composition at 90°C between pure MCH (α_{agg} = 0.46 at $c = 1 \times 10^{-4}$ M) and 15:85 v/v% $CHCl_3$-MCH (α_{agg} = 0.00 at $c = 1 \times 10^{-4}$ M) is not responsible for the resulting conformation of supramolecular polymers.

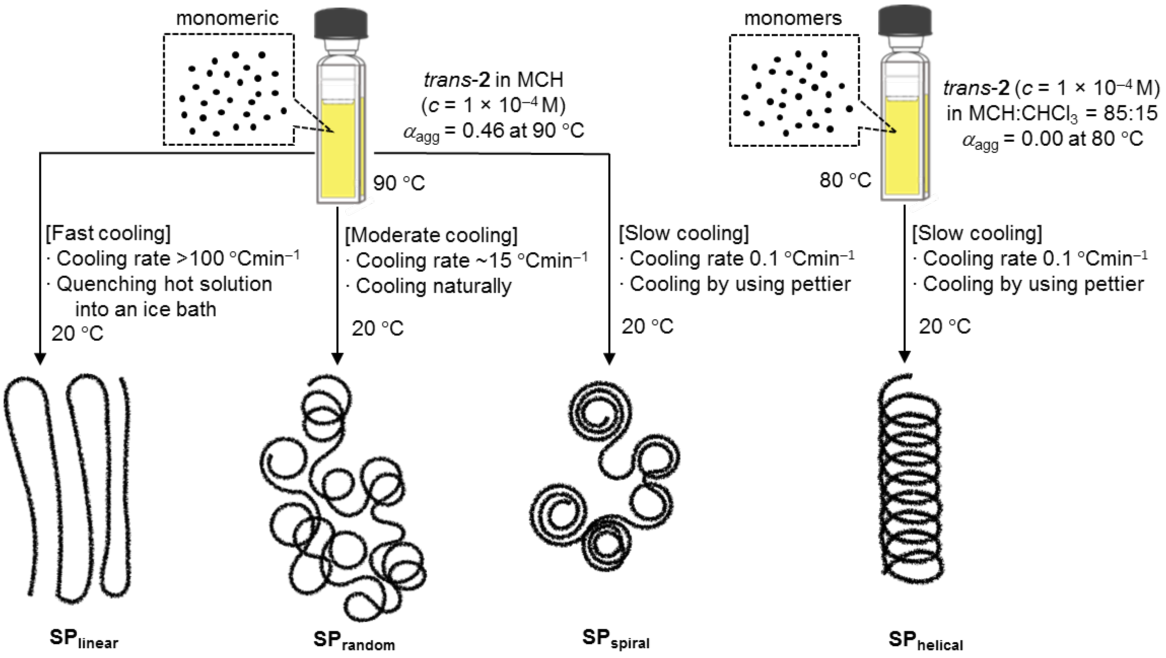

Supplementary Figure 22. Detailed schematic procedures for the preparation of supramolecular polymers.

Analysis of SAXS data. As mentioned in the main text, the samples **SP$_{\mathbf{random}}$** and **SP$_{\mathbf{spiral}}$** both exhibit maxima and minima in the SAXS data. In order to determine whether these might arise from the loops (average turning angle, $\theta_{av} > 360°$) apparent in the microscopy images, data analysis was carried out with models representing (a) toroids and (b) hollow cylinders. This approximation is used because to our knowledge a model describing the SAXS arising from a polymer chain containing multiple loops of similar size has not been reported, perhaps due to the rarity of such a structure. However, as shown in Supplementary Figure 5a, the model shapes (i.e. toroids or hollow cylinders) and the looped samples are similar. Given the reasonable quality of the fit and the good agreement between the values obtained by SAXS analysis and by AFM, it is highly likely that the

maxima/minima arise from the looped sample structure. The analysis models are described mathematically as follows:

The form factor for a torus with an elliptical cross-section, the scattering length density difference between the torus and the solvent $\Delta\rho$, radius R, cross-sectional radius a and aspect ratio b is generally given as follows[1,2]:

$$F_{torus}(Q,\Theta,R,a,b,\Delta\rho)=\int_{R-x}^{R+x}4\pi r\Delta\rho\frac{J_0(Qr\sin\Theta)\sin(Q\gamma(r)\cos\Theta)}{Q\cos\Theta}dr$$

(Supplementary Equation 1)

$$\text{where } \gamma(r)=b\sqrt{a^2-(r-R)^2}$$

(Supplementary Equation 2)

In equation (1), J_0 is the Bessel function of zero order. The overall scattering for a delta distribution of toroids with scale factor N is then given as:

(Supplementary Equation 3)

$$I_{torus}=N\int_0^{\pi/2}\left|F_{torus}(Q,\Theta,R,a,b,\Delta\rho)\right|^2\sin\Theta d\Theta$$

The form factor for a cylinder, with the scattering length density difference between cylinder and solvent $\Delta\rho$, radius R, length L is generally given as follows:[2]

$$K_{cyl}(Q,\Delta\rho,R,L,x)=2\pi R^2L\Delta\rho\frac{J_1\left(QR\sqrt{1-x^2}\right)}{QR\sqrt{1-x^2}}\frac{\sin\left(\frac{QLx}{2}\right)}{\frac{QLx}{2}}$$

(Supplementary Equation 4)

In equation (4), J_1 is the Bessel function of first order. The overall scattering for a delta distribution of hollow cylinders (ΔR = shell width) is then given as:

$$I_{hollow\,cyl}=N_{\mathrm{cyl}}\int_0^1\left(K_{cyl}(Q,\rho_{\mathrm{solv}}-\rho_2,R,L,x)+K_{cyl}(Q,\rho_2-\rho_{\mathrm{solv}},R+\Delta R,L,x)\right)^2dx$$

(Supplementary Equation 5)

In the above, ρ_{solv} and ρ_2 are the scattering length densities of the solvent and electron dense parts f 2, as described below. After R and ΔR had been found using the SASfit[2] analysis software, the

average loop center-to-center radii, r_{av} was calculated as $R + ½\ \Delta R$. In equation 5, N_{cyl} is a scale factor that accounts for the number density of toroids.

Finally, for the samples **SP$_{\mathbf{spiral}}$** and **SP$_{\mathbf{helical}}$** an additional peak function (Lorentzian) was needed to obtain an adequate fit, justified by the clear asymmetrical shape of the low Q maxima. This is given as follows:

$$f(Q, Q_0, \sigma, A) = \frac{A}{\pi}\left[\frac{\sigma}{(Q - Q_0)^2 + \sigma^2}\right] \quad \text{(Supplementary Equation 6)}$$

In the above, A is the peak amplitude, σ is the width and Q_0 is the peak centre. Judging from the microscopy images of **SP$_{\mathbf{helical}}$** and given the fitted position of peak centre (Q_0 = 0.499 nm^{-1}, so $d = 2\pi/Q$ = 12.6 nm), the peak may represent the repeat distance between spirals within the helices. However, for **SP$_{\mathbf{spiral}}$** it less clear what this feature (at Q_0 = 0.477 nm^{-1}, so $d = 2\pi/Q$ = 13.2 nm) in the scattering describes as no repeating distances of this length-scale are observed in the microscopy images. It may be that the structure of **SP$_{\mathbf{spiral}}$** is more unravelled in solution than when adsorbed on HOPG.

The scattering length density of the supramolecular polymer were approximated as arising solely from the electron dense parts of **2**, including the barbituric acid moiety, naphthalene moiety, azobenzene moiety and phenyl + methoxy groups. Considering only these groups, approximating the physical density (~1.3 g mL^{-1}) using the ACD ChemSketch plugin and inputting that into the NIST neutron activation and scattering calculator obtained a reasonable estimate of $\rho_2 = 12.5 \times 10^{-6}$ Å^{-2}. Using the same calculator, the scattering length density of methylcyclohexane ($\rho_{solv} = 7.5 \times 10^{-6}$ Å^{-2}) was found. These numbers were inputted into the SASfit software and held constant throughout the fitting process.

Supplementary Methods

General methods.

^{1}H and ^{13}C-NMR spectra were recorded on DPS300 (Bruker) or JNM-ECS500 (JEOL) NMR spectrometers, and chemical shifts are reported in ppm (δ) with the signal of TMS as an internal standard. ESI-MS spectra were measured on an Exactive (Thermo Scientific).

UV-Vis spectroscopy.

UV-Vis spectra were recorded on a JASCO V660 spectrophotometer with a Peltier device temperature-control unit. Screw-capped quartz cuvette (path length: 1 mm for 1×10^{-4} M solution; 10 mm for 2.5×10^{-5} M solution) was used for UV-Vis studies. UV-Vis measurements for light irradiation experiments were performed at a concentration (c) of 1×10^{-4} M in MCH. For temperature dependent UV-Vis experiments, the lower concentration ($c = 2.5 \times 10^{-5}$ M) was applied to obtain fully molecularly dissolved species at 90 °C, which is required for the plot of molar fractions of aggregated molecules (α_{agg}) against temperature. For AFM studies, the higher concentration ($c = 1 \times 10^{-4}$ M) was used to increase the population of self-assembled nanostructures. There was no significant morphological difference between supramolecular polymers prepared with two different concentrations (1×10^{-4} M and 2.5×10^{-5} M). Also, no significant differences in morphology were noticed when a 1×10^{-4} M MCH solution was cooling either from 90 °C or 100 °C.

Dynamic light scattering.

Dynamic light scattering measurements were conducted on Zetasizer Nano (Malvern Instruments). QS high precision cell (3 × 3 mm, Hellma Analytics) was used for the measurements. The temperature for measurements was kept at 20 °C.

Atomic force microscopy (AFM).

AFM images were obtained under ambient conditions using Multimode 8 Nanoscope V (Bruker Instrument) in Peak Force Tapping (Scanasyst) mode. Silicon cantilevers (SCANASYST-AIR) with a spring constant of 0.4 N/m and frequency of 70 kHz (nominal value, Bruker, Japan) were used. The samples were prepared by spin-coating (3000 rpm, for 1 min) of MCH solutions of supramolecular polymers onto freshly cleaved highly-oriented pyrolytic graphite (HOPG). 10 µL supramolecular polymer solution was injected on the HOPG (5 × 5 mm) for every measurement.

Transmission electron microscopy (TEM).

TEM images were acquired on JEM-2100F (JEOL) at an acceleration voltage of 120 kV. TEM samples were prepared by spin-coating of MCH solutions of supramolecular polymers onto

carbon-coated STEM Cu 75P grid (SHR-C075, grade: super ultrahigh resolution carbon, mesh 339, whole size 75 μm) and dried under air for 1 h followed by drying under vacuum for 24 h.

Synthesis and characterization of Compounds 2

Compounds **2** was prepared according to Supplementary Figure 23 given below.

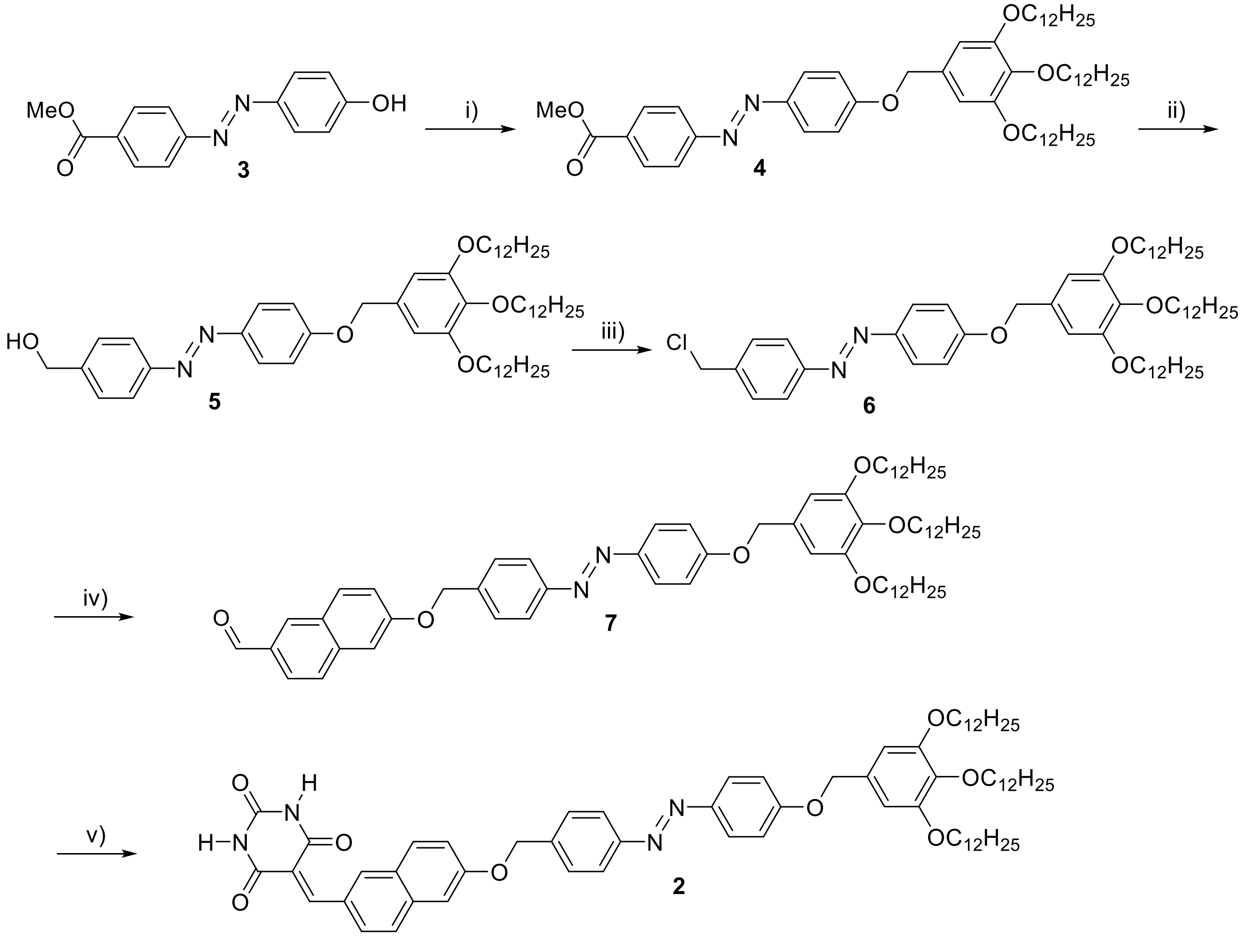

Supplementary Figure 23. Synthesis of **2**. i) 3,4,5-tri(*n*-dodecyloxy)benzyl chloride, K_2CO_3, dry *N,N*-dimethylformamide (DMF), 65 °C; ii) lithium aluminium hydride, dry diethylether; iii) $SOCl_2$, dry CH_2Cl_2, dry DMF; iv) 6-hidroxy-2-naphtaldehyde, K_2CO_3, dry DMF, 65 °C; v) barbituric acid, ethanol, reflux.

(*E*)-methyl 4-((4-((3,4,5-tris(dodecyloxy)benzyl)oxy)phenyl)diazenyl)benzoate (4): Compound **3**[3] (780 mg, 3.04 mmol) was dissolved in a suspension of K_2CO_3 (2.00 g, 14.5 mmol) in DMF (40 mL) at 65 °C, and the mixture was stirred for 10 min. 3,4,5-tri(*n*-dodecyloxy)benzyl chloride (2.04 g, 3.00 mmol) was added and the mixture was stirred for overnight at 65 °C. The

reaction mixture was poured into water and extracted with diethylether. The organic layer was dried over Na_2SO_4, concentrated in vacuo and evaporated to dryness. The resulting solid was recrystallized from acetone to afford **4** as orange solid (2.43 g, 90% yield). ^{1}H-NMR (300 MHz, $CDCl_3$): δ = 8.18 (d, J = 8.4 Hz, 2H), 7.96 (d, J = 9.0 Hz, 2H), 7.92 (d, J = 9.0 Hz, 2H), 7.11 (d, J = 9.0 Hz, 2H), 6.64 (s, 2H), 5.50 (s, 2H), 4.01–3.93 (m, 9H), 1.82–1.72 (m, 6H), 1.55–1.26 (m, 54H), 0.90–0.85 (m, 9H). ^{13}C-NMR (125 MHz, $CDCl_3$): δ = 166.7, 161.9, 155.4, 153.5, 147.2, 138.2, 131.3, 131.2, 130.7, 125.3, 122.5, 115.3, 106.2, 73.6, 70.8, 69.2, 52.4, 31.9, 29.7, 29.4, 26.1, 22.8, 14.1; MS (ESI) : *m/z* calcd for $C_{57}H_{91}O_6N_2$ = 899.6872 $[M+H]^+$, found 899.6866.

(*E*)-(4-((4-((3,4,5-tris(dodecyloxy)benzyl)oxy)phenyl)diazenyl)phenyl)methanol (5): Compound **4** (2.43 g, 2.70 mmol) was dissolved in dry diethylether (40 mL) at room temperature. Lithium aluminium hydride (105 mg, 2.77 mmol) was added slowly and the mixture was stirred for 3 h at room temperature. The reaction mixture was added dropwise into water and washed with diethylether. The organic layer was dried over Na_2SO_4, concentrated in vacuo and evaporated to dryness to afford pure **5** as yellow solid without purification (1.95 g, 83% yield). ^{1}H-NMR (300 MHz, $CDCl_3$): δ = 7.94 –7.87 (m, 4H), 7.50 (d, J = 9.0 Hz, 2H), 7.10 (d, J = 9.0 Hz, 2H), 6.64 (s, 2H), 5.03 (s, 2H), 4.79 (s, 2H), 4.01–3.93 (m, 6H), 1.82–1.72 (m, 6H), 1.55–1.26 (m, 54H), 0.90–0.86 (m, 9H). [Note: the alcoholic proton is not observed] ^{13}C-NMR (125 MHz, $CDCl_3$): δ =161.2, 153.4, 152.2, 147.1, 143.2, 138.1, 131.2, 127.4, 124.7, 122.8, 115.1, 106.1, 73.5, 70.7, 69.1, 64.9, 31.9, 29.6, 29.4, 26.1, 22.7, 14.1; MS (ESI) : *m/z* calcd for $C_{56}H_{91}O_5N_2$ = 871.6923 $[M+H]^+$, found 871.6918.

(*E*)-1-(4-(chloromethyl)phenyl)-2-(4-((3,4,5-tris(dodecyloxy)benzyl)oxy)phenyl)diazene (6): Compound **5** (1.65 g, 2.24 mmol) was dissolved in a mixture of dry CH_2Cl_2 (30 mL) and dry DMF (0.2 mL). $SOCl_2$ (1.0 mL) was added and the mixture was stirred for 4 h at room temperature. The resultant solution was poured into saturated aqueous $NaHCO_3$ solution and extracted with diethylether. The organic layer was dried over Na_2SO_4, concentrated in vacuo and evaporated to dryness. The residue was purified by column chromatography on silica gel (hexane/AcOEt = 9:1) to give compound **6** as white solid (1.66 g, 83%). ^{1}H-NMR (300 MHz, $CDCl_3$): δ = 7.94–7.86 (m, 4H), 7.52 (d, J = 8.4 Hz, 2H), 7.09 (d, J = 9.0 Hz, 2H), 6.64 (s, 2H), 5.04 (s, 2H), 4.66 (s, 2H), 4.01–3.93 (m, 6H), 1.82–1.72 (m, 6H), 1.55–1.26 (m, 54H), 0.90–0.86 (m, 9H). ^{13}C-NMR (125 MHz, $CDCl_3$): δ = 161.4, 153.4, 152.5, 147.1, 139.5, 138.1, 131.2, 129.3, 12.3, 1244.8, 122.9, 115.1, 73.4, 70.7, 69.1, 45.8, 32.9, 29.6, 29.4, 26.1, 22.8, 14.2; MS (ESI): *m/z* calcd for $C_{56}H_{90}O_4N_2Cl$ = 889.6584 $[M+H]^+$, found 889.6576.

(*E*)-6-((4-((4-((3,4,5-tris(dodecyloxy)benzyl)oxy)phenyl)diazenyl)benzyl)oxy)-2-naphthaldehyde (7): 6-Hidroxy-2-naphtaldehyde (40 mg, 0.232 mmol) was dissolved in a suspension of K_2CO_3 (360 mg, 2.76 mmol) in DMF (30 mL) at 65 °C, and the mixture was stirred until the solution turned green. Compound **6** (200 mg, 0.225 mmol) dissolved in DMF was added dropwise and the mixture was stirred for 5 h at 65 °C under N_2 atmosphere. The reaction mixture was poured into water and extracted with diethylether. The organic layers was dried over Na_2SO_4, concentrated in vacuo and evaporated to dryness. The residue was purified by column

chromatography on silica gel (hexane/AcOEt = 4:1) to give compound **7** as orange solid (112 mg, 46% yield). ^{1}H-NMR (300 MHz, $CDCl_3$): δ = 10.11 (s, 1H), 8.28 (s, 1H), 7.94 (d, J = 9.0 Hz, 6H), 7.81 (d, J = 8.4 Hz, 1H), 7.63 (d, J = 8.4 Hz, 2H), 7.37–7.33 (m, 1H), 7.29 (m, 1H), 7.09 (d, J = 9.0 Hz, 2H), 6.64 (s, 2H), 5.30 (s, 2H), 5.04 (s, 2H), 4.01–3.93 (m, 6H), 1.83–1.72 (m, 6H), 1.47–1.26 (m, 54H), 0.90–0.86 (m, 9H). ^{13}C-NMR (125 MHz, $CDCl_3$): δ = 192.0, 161.5, 159.2, 153.4, 152.6, 147.1, 138.5, 138.12, 138.1, 134.2, 132.5, 131.3, 128.1, 127.8, 124.8, 123.7, 122.9, 120.2, 115.1, 107.5, 106.1, 73.5, 70.7, 69.8, 69.1, 31.9, 30.3, 29.6, 26.1, 22.7, 14.1; MS (ESI): *m/z* calcd for $C_{67}H_{96}O_6N_2Na$ = 1047.7161 $[M+Na]^+$, found 1047.7148.

(*E*)-5-((6-((4-((4-((3,4,5-tris(dodecyloxy)benzyl)oxy)phenyl)diazenyl)benzyl)oxy)naphthalen-2-yl)methylene)pyrimidine-2,4,6(1H,3H,5H)-trione (2): Compound **7** (83 mg, 0.0809 mmol) was dissolved in ethanol (40 mL), and stirred until the solution turned clear at 80°C with reflux. Barbituric acid (86 mg, 0.25 mmol) was added and the mixture was stirred for 4.5 h at 80 °C under reflux. The reaction mixture was cooled to room temperature and the resulting precipitates were collected by filtration, washed by hot ethanol to give pure compound **1** as yellowish solid (87 mg, 95%).

NMR (500 MHz, $CDCl_3$, 1.5 mM): δ = 8.77–8.71 (d, 2H), δ = 8.33–8.31 (d, 1H), δ = 7.99 (s, 1H), δ = 7.93 – 7.90 (m, 5H), δ = 7.87 (s, 1H), δ = 7.76–7.75 (d, 1H), δ = 7.62–7.60 (d, 2H), δ = 7.32–7.30 (d, 1H), δ = 7.25 (s, 1H), δ = 7.09–7.07 (d, 2H), δ = 6.63 (s, 2H), δ = 5.30 (s, 2H), δ = 5.03 (s, 2H), δ = 3.99–3.93 (m, 6H), δ = 1.80–1.74 (m, 6H), δ = 1.56–1.25 (m, 54H), δ = 0.88–0.85 (m, 9H). ^{13}C-NMR (125 MHz, $CDCl_3$): δ = 192.00, 161.36, 159.18, 153.35, 152.55, 147.08, 134.23, 131.28, 128.08, 124.81, 123.69, 122.90, 120.19, 115.11, 107.51, 106.13, 73.45, 70.69, 69.75, 31.92, 30.33, 29.69, 26.09, 22.69, 14.12; MS (ESI) : *m/z* calculated for $C_{71}H_{99}O_8N_4$ = 1135.7457 $[M+H]^+$, found 1135.7457.

Supplementary References

1. Kawaguchi, T. Radii of gyration and scattering functions of a torus and its derivatives. *J. Appl. Cryst.* **34**, 580–584 (2001).

2. Breßler, I. Kohlbrecher & J. Thünemann, A. SASfit: a tool for small-angle scattering data analysis using a library of analytical expressions. *J. Appl. Cryst.* **48**, 1587–1598 (2015).

3. Zhang, W. Xie, J. Yang, Z. Shi, W. Aggregation behaviors and photoresponsive properties of azobenzene constructed phosphate dendrimers. *Polymer* **48**, 4466–4481 (2007).
